# The Effects of a Web-Based Tool for Parents of Children With Juvenile Idiopathic Arthritis: Randomized Controlled Trial

**DOI:** 10.2196/29787

**Published:** 2022-05-12

**Authors:** Kathleen Mulligan, Shashivadan P Hirani, Sally Harris, Jo Taylor, Lucy R Wedderburn, Stanton Newman, WebParC Investigator group

**Affiliations:** 1 Centre for Health Services Research, School of Health Sciences City, University of London London United Kingdom; 2 East London NHS Foundation Trust London United Kingdom; 3 Brighton and Sussex University Hospitals NHS Trust Brighton United Kingdom; 4 University College London Great Ormond Street Institute of Child Health University College London London United Kingdom; 5 Centre for Adolescent Rheumatology Versus Arthritis London United Kingdom; 6 National Institute for Health Research Biomedical Research Centre at Great Ormond Street Hospital London United Kingdom; 7 See Acknowledgments

**Keywords:** parenting stress, juvenile idiopathic arthritis, web-based intervention, randomized controlled trial, parenting, pediatrics, arthritis, RCT, rheumatology, children, youth, web-based tool, mobile phone

## Abstract

**Background:**

Juvenile idiopathic arthritis (JIA) is a group of autoinflammatory diseases that cause pain and disability if not controlled by treatment. Parenting a child with JIA is stressful for parents, who express concerns about their child’s treatment and may experience anxiety and powerlessness concerning their child’s illness. Parenting stress is greater in parents of children with chronic illness than in those with healthy children and is related to poorer psychological adjustment in both parents and children. It is therefore important to develop interventions to support parents. This paper reports the evaluation of a web-based tool that provides information and practical skills to help increase parents’ confidence in managing their child’s illness and reduce parenting stress.

**Objective:**

The aim of this study is to evaluate the benefits of a web-based tool (*WebParC*) for parents of children with recently diagnosed JIA.

**Methods:**

A multicentered randomized controlled trial was conducted at pediatric rheumatology centers in England. We recruited parents of children aged ≤12 years who had been diagnosed with JIA within the previous 6 months. They were randomized to the intervention (WebParC access plus standard care) or the control (standard care alone) and followed up 4 months and 12 months after randomization. Where both parents participated, they were randomized *by household* to the same trial arm. The WebParC intervention consists of information about JIA and its treatment plus a toolkit, based on cognitive behavioral therapy, to help parents develop skills to manage JIA-related issues. The primary outcome was the self-report Pediatric Inventory for Parents measure of illness-related parenting stress. The secondary outcomes were parental mood, self-efficacy, coping, effectiveness of participation in their child’s health care, satisfaction with health care, and child’s health-related quality of life.

**Results:**

A total of 203 *households* comprising 220 parents were randomized to the intervention (100/203, 49.3%) or control (103/203, 50.7%) arm. Follow-up assessments were completed by 65.5% (133/203) of the households at 4 months (intervention 60/100, 60%, and control 73/103, 70.9%) and 61.1% (124/203) of the households at 12 months (intervention 58/100, 58%, and control 66/103, 64.1%). A main effect of the trial arm was found on the Pediatric Inventory for Parents: the intervention participants reported less frequency (subscales *communication F*_1,120627_=5.37; *P*=.02, and *role function F*_1,27203_=5.40; *P*=.02) and difficulty (subscales *communication F*_1,2237_=7.43; *P*=.006, *medical care F*_1,2907_=4.04; *P*=.04, and *role function F*_1,821_=4.37, *P*=.04) regarding illness-related stressful events than the control participants.

**Conclusions:**

The WebParC website for parents of children with JIA reduced illness-related parenting stress. This web-based intervention offers a feasible preventive approach for parents of children with JIA and potentially could be adapted and evaluated for parents of children with other chronic illnesses.

**Trial Registration:**

International Standard Randomized Controlled Trial Number (ISRCTN) 13159730; http://www.isrctn.com/ISRCTN13159730

## Introduction

### Background

Parenting a child with juvenile idiopathic arthritis (JIA) presents many challenges, including dealing with the child’s pain, distress, and physical difficulties; managing medication, hospital visits (which may involve traveling a considerable distance from home), and impact on schooling; financial issues such as time off work; and uncertainty about the future. In addition, in some health care systems, there are substantial medication costs. Parents of children with JIA have concerns about their child’s treatment [1-3] and may experience anxiety and powerlessness concerning their child’s illness [4]. Parenting stress is greater in parents of children with chronic illness than in those with healthy children [5] and is associated with poorer psychological adjustment in both parents and children [5,6]. Given the interconnectedness between parent and child adjustment, early intervention to support parents may facilitate better adjustment for their children with JIA [7,8]. The Pediatric Psychosocial Preventative Health Model developed by Kazak [8] is a 3-tier model for treating the families of children in pediatric health settings. The model offers a guide for matching psychosocial support to families’ level of need. It proposes that most families of children with chronic illnesses are likely to be distressed but resilient (universal tier). A smaller group of families have risk factors for ongoing difficulties and require targeted care. The smallest group, clinical/treatment, has a high level of risk factors for ongoing distress and requires more intensive clinical services. Kazak [8] stresses the need to adopt preventive approaches to support families in the universal tier to build their resilience and prevent future problems.

A potential preventive approach is to provide web-based interventions. It is important that parents are able to access information from a trusted source [9]; however, health information on the internet is unregulated, often not validated through a systematic process [10], and the quality is variable [11,12]. When developing this research, none of the websites providing information for children and young people with JIA and their parents had been evaluated in a randomized controlled trial (RCT) and none provided skills training in techniques to help parents to manage their child’s arthritis [12].

We developed a website for parents of children with recently diagnosed JIA (*WebParC*) [13] to complement usual clinical care, with potential to help parents cope with the stress of their child having JIA. It is a specially designed web-based tool providing around-the-clock access to information and practical skills in dealing with specific problems (eg, taking medication) and accessible as need arises. This paper reports the evaluation of WebParC.

### Objectives

The aim of the study is to test the hypothesis that parents provided with WebParC in addition to standard care would experience less illness-related parenting stress than those provided standard care alone.

## Methods

### Design

This was a multicenter RCT.

### Participating Research Sites

A total of 16 National Health Service tertiary pediatric rheumatology services in England participated in the study.

### Ethical Approval

Approval was obtained from the Health Research Authority London Bridge Research Ethics Committee (13/LO/0288).

### Research Participants

The participants were parents attending rheumatology clinic appointments with their child, who met the criteria outlined in Textbox 1.

**Inclusion and exclusion criteria.**

**Inclusion criteria**

- Parent aged ≥18 years, with a child aged ≤12 years, recently diagnosed with juvenile idiopathic arthritis (within the previous 6 months)
  It was considered appropriate to focus the website on this age group because responsibility is taken mainly by parents in the child’s earlier years but tends to move to the child as they get older; therefore, different strategies may be required for parents of adolescents
  Juvenile idiopathic arthritis was diagnosed by a pediatric rheumatologist according to current International League of Associations for Rheumatology criteria [14], which specify that juvenile idiopathic arthritis involves inflammation of the joints that begins before the age of 16 years and persists for at least 6 weeks. The International League of Associations for Rheumatology categorizes 7 juvenile idiopathic arthritis subtypes that differ in clinical course and are based on the number of inflamed joints, laboratory tests, and clinical features. The subtypes are oligoarticular, polyarticular–rheumatoid factor negative, polyarticular–rheumatoid factor positive, systemic-onset, psoriatic, enthesitis-related, and undifferentiated arthritis
  One or both parents could participate. Parents did not need to be living together or with the child with juvenile idiopathic arthritis to be eligible
- Internet access
- Able to speak and read English

**Exclusion criteria**

- Current severe mental illness such as identifiable psychosis in parents
- Major problems with literacy, making the questionnaire completion impossible
- Likely to be distressed by the study, as judged by their child’s rheumatologist

### Procedures

#### Overview

Parents were invited to participate by their child’s rheumatologist when attending a clinic appointment with their child. Interested parents were given the opportunity to ask questions and were given the participant information sheet to read at the clinic or to take home if they wished to have more time to consider participation. Those who wished to participate provided written consent to the site research staff. If the child with JIA was aged 8-12 years, the child’s assent was obtained for their demographic and clinical data to be collected for the research. After providing consent, participants were given baseline questionnaires to complete at the clinic or at home and return them to the trial coordinating center (a freepost envelope was provided). A link to a web-based version of the questionnaire on Qualtrics software was also provided so that parents could choose their preferred completion mode. Where both parents participated, they were given questionnaire packs with separate return envelopes and individual study IDs that they entered into Qualtrics if they chose web-based completion. If the baseline questionnaire was not returned, a member of the site research team contacted the participants by telephone. This was a change to the protocol made partway through the trial to enhance questionnaire return rates.

#### Randomization

To minimize selection bias, participants were randomized by the trial coordinating center after receipt of the completed baseline questionnaire. Randomization was in a ratio of 1:1 to trial arms. Where both parents participated, they were randomized to the same trial arm (ie, randomization was clustered by *household*). Blocked randomization per site was performed using computer-generated randomization sequences that allowed varying randomization block sizes. A combination of block sizes was used, varying among 2, 4, and 6, depending on site size; we used different-sized blocks so that sites could not guess which group the last participant of a block would be randomized to. Allocation was concealed from clinical teams to avoid biasing clinical care; however, after allocation, it was not possible to blind the trial coordinator because the follow-up questionnaires contained additional questions about the website for intervention arm participants. Other members of the investigating team were blinded to trial arm allocation. Participants were requested not to inform their child’s clinicians of their trial arm allocation.

#### Trial Arms

##### Control Arm

Children of control arm participants continued to receive standard clinical care as provided by the study site.

##### Intervention Arm

In addition to standard care, those allocated to the intervention arm were given free unlimited password-protected access to the website.

### Website Design

The *JIA website for Parents* site was designed following:

1. A review was conducted of the literature on parents’ experiences of having a child with JIA.
2. A review of websites was conducted to find those that (1) included information about JIA for parents, (2) provided specific skills training for parents to manage their child’s JIA, and (3) contained information in English. Although many sites were found that provided information about JIA, at the time of review, 5 main websites [15-19] that contained significant information for parents were reviewed in detail but none contained skills training to assist parents.
3. A focus group was conducted with 6 parents to ask their views on what the website should include.
4. We conducted 2 focus groups with 12 health care professionals—6 (50%) rheumatologists, 5 (42%) rheumatology nurse specialists, and 1 (8%) clinical psychologist—to ask their views on what the website should include.

Website content was written by health professionals supported by a research assistant. The health professionals included 13 rheumatologists, 4 rheumatology nurse specialists, 2 clinical psychologists, an occupational therapist, an ophthalmologist, 2 physiotherapists, a podiatrist, and a social worker. A website consultant designed the site for layout, usability, and acceptability.

The resulting prototype website was tested by 7 parents and eight health professionals (4, 50%, rheumatologists; 2, 25%, rheumatology nurse specialists; 1, 13%, physiotherapist; and 1, 13%, clinical psychologist) to evaluate usability, navigation, structure, layout, and content. Minor changes were made to the website after this assessment. These included condensing some of the text, improving some text formatting with the use of bullet points, correcting a few navigation links, and renaming some tabs with more user-friendly terms.

Over the course of the website development but before trial commencement, the website was reviewed and updated to ensure that user interfaces and content were current. Website content did not change thereafter during the trial period. The website is device adaptive; therefore, it is suitable for use on computer, tablet, and smartphone.

The website has two main components:

1. Information about JIA and its treatment. This comprises sections about cause, diagnosis, JIA types and symptoms, how JIA changes with time, possible complications, the rheumatology team, and everyday life and available treatments. It also includes videos of health professionals explaining JIA and its treatment as well as video testimonials from parents about living with, and caring for, children with JIA as a family.
2. A JIA toolkit based on cognitive behavioral therapy that includes psychoeducation about thoughts, feelings, and behavior following a diagnosis; cognitive restructuring techniques to challenge unhelpful thinking to promote coping with JIA; problem-solving skills to promote coping with adherence issues and stressful events; strategies to promote effective communication with family members and the health care team; and pain management techniques, including cognitive restructuring, relaxation, distraction, and pacing.

The toolkit includes a number of downloadable resources such as problem-solving sheet, thought diary, breaking negative thought cycle sheet, reward chart, procedure contract template and certificate, visual timetable, and audio relaxation sessions for children and adults.

### Trial Measures

#### Parent Data

Parents provided demographic data including age, gender, education level, and relationship status.

Information on the validated self-report questionnaire measures is reported in Table 1. The primary outcome was parenting stress at 4 months after randomization, measured with the Pediatric Inventory for Parents (PIP) [20], which is a validated measure to assess difficult events that parents may face. Respondents answer two questions for each event: how often it occurred in the past 7 days and how difficult it was for them. The 4-month time span was chosen to give parents sufficient time to make use of the website and to evaluate its effect in the short term. Follow-up times were also selected to fit around clinic visits.

The secondary outcomes were as follows:

- Parenting stress at 12 months after randomization using the PIP [20]. This time span was chosen to evaluate the medium-term effects of using the website.
- Parent mood, assessed with the Hospital Anxiety and Depression Scale [21].
- Parent confidence in managing their child’s arthritis, assessed with the Parent’s Arthritis Self-Efficacy Scale (PASE) [22].
- Parent effectiveness in managing their child’s health care, assessed using the Effective Consumer Scale–Adapted (ECS17-A) [23]. The original scale developed for adults with musculoskeletal disease was adapted to refer to how parents manage their child’s disease.
- Parent satisfaction with health care, assessed with the Client Satisfaction Questionnaire [24].
- A proxy measure of the child’s health-related quality of life was assessed with the Child Health Questionnaire, 50-item parent version (CHQ-PF50) [25].

Process measures on website use and parent coping and beliefs about their child’s illness (Brief Coping Orientation to Problems Experienced [26] and Brief Illness Perception Questionnaire [27], respectively) were collected but will be reported separately from this paper, which focuses on the trial outcomes.

**Table 1:** Trial measures.

Measure and subscales			Number of items	Response scale	Scoring	Cronbach *α* in WebParC study at baseline
**Pediatric Inventory for Parents [20]**				1=never to 5=very often and 1=not at all to 5=extremely	Higher score=greater frequency or difficulty of stressful events	
	2 subscales (Frequency and Difficulty)		84 (42 for each subscale)		Total 42-210 in each subscale	
	**4 domains**				Frequency total score: .959; Difficulty total score: .965	
		Communication	9		9-45	Frequency: .787; Difficulty: .841
		Emotional distress	15		15-75	Frequency: .918; Difficulty: .913
		Medical care	8		8-40	Frequency: .840; Difficulty: .846
		Role function	10		10-50	Frequency: .840; Difficulty: .864
**Hospital Anxiety and Depression Scale [21]**						
	Anxiety and Depression		7 items per scale	0-3 (response options vary for each item)	0-21 per subscale (mild: 8-10; moderate: 11-14; severe: 15-21); higher score=more symptoms of anxiety or depression	Anxiety: .900; Depression: .872
**Parent’s Arthritis Self-Efficacy Scale [22]**						
	Symptoms and Psychosocial		14 (7 in each subscale)	1=very uncertain to 7=very certain or not applicable	Scores are standardized to 0-10 for each subscale. Higher score=greater self-efficacy	Symptoms: .902; Psychosocial: .934
**Effective Consumer Scale–Adapted [23]**			17	0=never to 4=always	A score for each domain and a total score are calculated and converted to 0-100. Higher score=a more effective consumer of health care	Total score: .933
	Use of health information		3			.812
	Clarifying personal priorities		3			.836
	Communicating with others		3			.801
	Negotiating roles and taking control		4			.798
	Deciding and taking action		4			.876
**Client Satisfaction Questionnaire [24]**						
	N/A^a^		8	1-4 (response options vary for each item)	A total score is calculated. Higher score=greater satisfaction with healthcare	.898
**Child Health Questionnaire 50-item parent version [25]**			50	Variable	Standardized to population norms and range from 0 to 100 (mean 50, SD 10). Higher score=better health-related quality of life	
	**Summary scores**					N/A
		Physical				
		Psychosocial				
	**Subscales**					
		Physical functioning				.942
		Role/social limitations–emotional/behavioral				.988
		Role/social limitations–physical				.962
		Bodily pain and discomfort				.923
		Behavior				.905
		Mental health				.921
		Self-esteem				.954
		General health perceptions				.709
		Parental impact–emotional				.821
		Parental impact–time				.807
		Family activities				.940
		Family cohesion				N/A: single item

^a^N/A: not applicable.

#### Child Data

Information about participants’ children with JIA (gender, age, JIA subtype, date of diagnosis, core outcome variables (number of inflamed and limited joints, erythrocyte sedimentation rate, Child Health Assessment Questionnaire, parent global rating, and physician global rating) [28], medication, and any related comorbidities were gathered by the clinical sites and sent securely to the trial coordinating center.

### Follow-up

At 4 and 12 months after randomization, the trial center sent follow-up questionnaires both electronically and in hard copy for participants to choose their preferred completion method. Where both parents participated, they were mailed the follow-up questionnaire packs individually and a link to the web-based questionnaire was sent to their individual email addresses. Up to two telephone or text reminders were sent if questionnaires were not returned within 2 weeks. Follow-up clinical data for the child (core outcome variables, medication, and comorbidities) were obtained from trial sites’ clinic notes closest to the follow-up time points.

Participants were sent a £5 (US $6.80) gift voucher on return of each completed study questionnaire. This protocol change was made partway through the trial to enhance questionnaire return rates, but the gift voucher was offered retrospectively to all participants.

### Statistical Considerations

#### Sample Size

Both parents were invited to participate; therefore, sample size calculation allowed for *clustering* by household. The power calculation was based on the PIP primary outcome measure, assessed at 4 months after randomization. SDs on the PIP scales frequency (PIP-F) and difficulty (PIP-D) were expected to be 25 [20]. Therefore, 85 households per trial arm was considered adequate to detect a mean difference of 10 points with 80% power and 5% significance level, representing a medium effect size. This allowed for clustering by household, assuming an intracluster correlation of 0.5. Allowing for a 15% dropout rate, 100 households per trial arm (200 total) were needed.

#### Statistical Analysis

Data were collected and stored in a secure manner in accordance with the guidelines of the United Kingdom’s Data Protection Act and the European Union’s General Data Protection Regulation. Analysis was conducted using SPSS software (version 25.0; IBM Corp).

Missing value analysis examined item-level missing data. Scale authors’ rules, where available, were applied for dealing with missing data. If rules were not available, mean imputation within a scale was used when ≥50% of the scale items were available. The Little missing completely at random (MCAR) test was conducted to indicate the appropriateness of further imputations. If the levels of missing data on any scale or item were >10%, multiple imputation was conducted (m=10). Data from all time points (baseline, 4 months, and 12 months) were used to predict missing data, but the 3 time points were imputed separately and only for participants who provided data at that time point. Resultant databases were analyzed separately, after which Rubin’s rules [29,30] were used to combine the results from the 10 data sets. Responders (those who completed at least one follow-up) were compared with nonresponders on baseline characteristics using logistic regression analyses.

Analyses were on an intention-to-treat basis. Outcomes were compared using multilevel modeling with a random effect of household and adjusting for the variable at baseline and any parent and child demographic characteristics that differed between trial arms. We explored the main effects of time and trial arm and their interaction by entering trial arm, time, and the interaction between trial arm and time as fixed effects, using the restricted maximum likelihood estimation method. Significant interaction terms were interpreted as indicating differential treatment effectiveness and explored with post hoc tests. Hedges *g* was calculated for effect sizes of differences between trial arms at each follow-up.

## Results

### Overview

**Figure 1:**
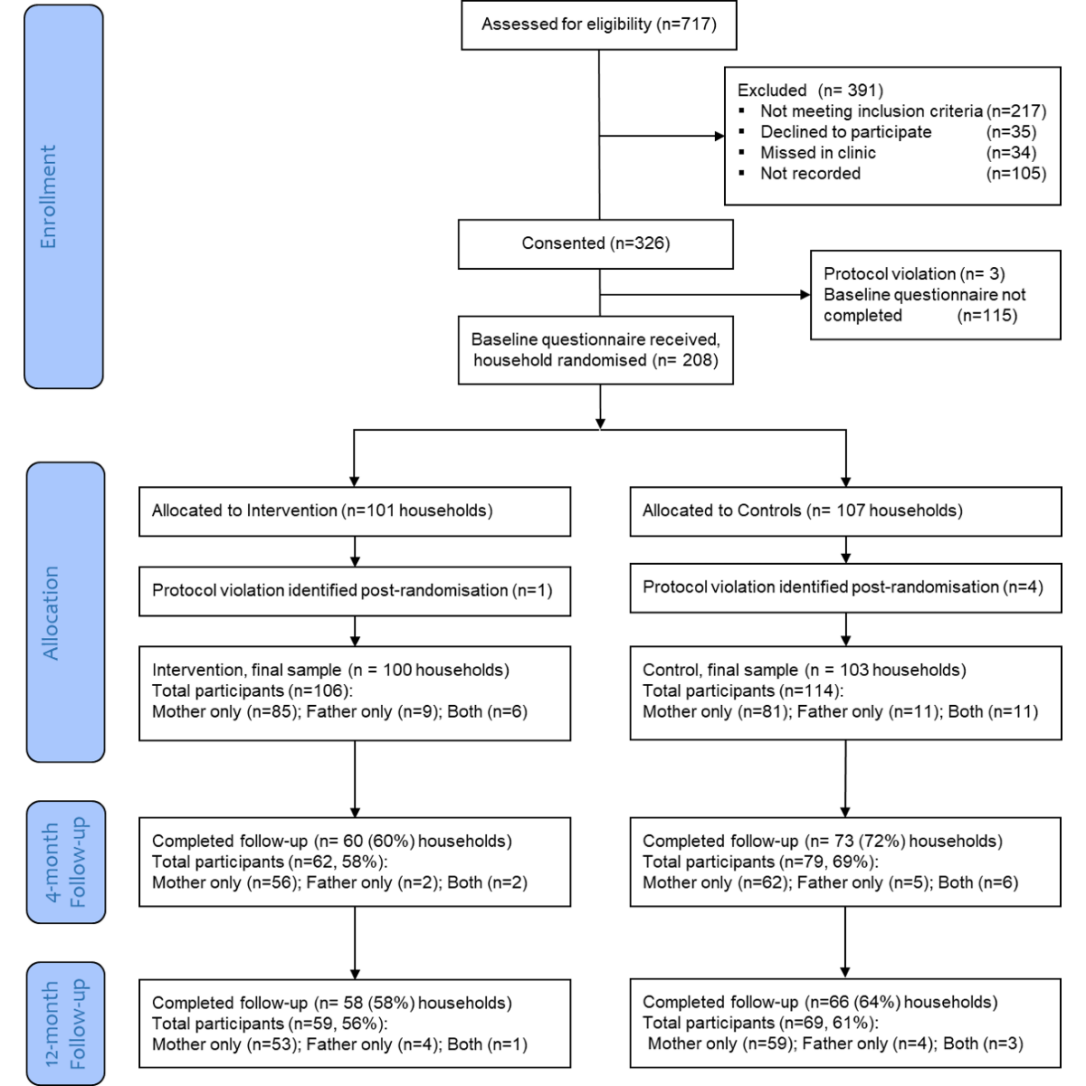
CONSORT (Consolidated Standards of Reporting Trials) flow diagram.

Between February 2016 and October 2018, 717 parents were assessed for eligibility and 326 (45.5%) consented to take part. Baseline questionnaires were returned by parents of 207 children (*households*). In all, 4 protocol violations were identified (diagnosis not JIA, 2/4, 50%; >6 months since diagnosis, 1/4, 25%; and consent form not received, 1/4, 25%), leaving a final sample of 203 households (220 parents), 100 (49.3%) households (106/220, 48.2%, parents) randomized to the intervention arm and 103 (50.7%) households (114/220, 51.8%, parents) randomized to the control arm (Figure 1). Follow-up questionnaires were completed by 65.5% (133/203) of the households (141/220, 64.1%, parents) at 4 months and 61.1% (124/203) of the households (128/220, 58.2%, parents) at 12 months. Attrition did not differ significantly between intervention and control at 4 months (*χ*^2^_1_=2.8; *P*=.10) or 12 months (*χ*^2^_1_=0.5; *P*=.47). Responders (those who completed one or both follow-ups) differed from nonresponders (those who completed neither follow-up) on two baseline variables: mothers (146/183, 79.8%) responded proportionally more than fathers (20/37, 54.1%; B=1.210, SE 0.378; *P*=.001; odds ratio 3.354, 95% CI 1.600-7.033) and responders scored higher on the baseline ECS17-A subscale *use of health information* (mean 77.4, SE 1.26) than nonresponders (mean 71.0, SE 2.77; B=0.020, SE 0.009; *P*=.02; odds ratio 1.021, 95% CI 1.003-1.039).

### Missing Values

At baseline, 58.8% (50/85) of the variables and 88.2% (194/220) of the cases had complete data, relating to an overall missing data level of 5.7% (Little MCAR test: *χ*^2^_7726_=7356.6; *P*=.99). At the 4-month follow-up, 83.7% (118/141) of the cases followed up had complete data but no variables were complete, relating to an overall missing data level of 9.4% (Little MCAR test: *χ*^2^_3333_=454.2; *P*=.99). At the 12-month follow-up, 82.8% (106/128) of the cases had complete data, with 87% (40/46) of the variables having complete data, relating to an overall missing data level of 12.4% (Little MCAR test: *χ*^2^_2978_=140.3; *P*=.99).

### Baseline Characteristics

Baseline characteristics are shown in Table 2. In 8.4% (17/203) of the cases, both parents took part. Most of the participants were mothers (183/220, 83.2%). The parents’ average age was 36.5 (SD 6.5) years, and 31.8% (70/220) were educated to degree level or above. Participants’ children with JIA were predominantly girls (136/203, 67%), with a mean age of 6.1 (SD 3.4) years. The most frequent JIA subtypes were oligoarticular (107/203, 52.7%) and polyarticular (65/203, 32%).

Unadjusted means and SEs for all questionnaires at each time point are presented in Multimedia Appendix 1. No difference between the trial arms was found on any clinical or self-report questionnaire at baseline; however, education level was higher in the intervention arm (mean 3.65, SD 1.63; control: mean 3.17, SD 1.52; *F*_1,218_=5.221; *P*=.02). This was controlled for in all analyses.

**Table 2:** Participant characteristics at baseline.

							Intervention		Control						Total
**Parents, details**															
	Households, n						100		103						203
	Participants, n						106		114						220
	**Parents, n (%)**														
		Mother				91 (85.8)		92 (80.7)						183 (83.2)	
		Father				15 (14.2)		22 (19.3)						37 (16.8)	
	Age (years), mean (SD)						35.8 (6.5)		37.2 (6.4)						36.5 (6.5)
	**Education, n (%)**														
		≤GCSE^a^ or equivalent		33 (31.2)							52 (45.6)		85 (38.7)		
		Advanced Level^b^ or equivalent		26 (24.5)							25 (21.9)		51 (23.2)		
		HNC^c^ or HND^d^		7 (6.6)							7 (6.1)		14 (6.4)		
		Degree or postgraduate		40 (37.7)							30 (26.3)		70 (31.8)		
	**Relationship status, n (%)**														
		Single or divorced or separated		11 (10.4)							19 (16.6)		30 (13.6)		
		Married or living with partner or in a relationship		95 (89.6)							95 (83.3)		190 (86.4)		
	Living with child with JIA^e^, n (%)						104 (98.1)		111 (97.4)						215 (97.7)
	Average number of children per family, mean (SE)						2.11 (0.09)		2.03 (0.09)						2.07 (0.06)
**Child with** **JIA,** **details**															
		Total, n				100		103						203	
		**Gender, n (%)**													
			Female			69 (69)		67 (65)						136 (67)	
			Male			31 (31)		36 (35)						67 (33)	
	Age (years), mean (SD)						6.3 (3.2)		6.0 (3.7)						6.1 (3.4)
	**JIA** **subtype, n (%)**														
		Systemic				4 (4)		4 (3.9)						8 (3.9)	
		Oligoarticular				58 (58)		49 (47.6)						107 (52.7)	
		Polyarticular				28 (28)		37 (35.9)						65 (32)	
		Psoriatic				6 (6)		5 (4.9)						11 (5.4)	
		ERA^f^				3 (3)		4 (3.9)						7 (3.4)	
		Undifferentiated				1 (1)		4 (3.9)						5 (2.5)	
	**Current disease severity, median (IQR)**														
		Number of active joints (known for 189/203, 93.1%)				2 (1-5)		2 (1-5.5)						2 (1-5)	
		Number of limited joints (known for 189/203, 93.1%)				2 (1-4)		2 (1-4)						2 (1-4)	
		CHAQ^g^ (known for 128/203, 63.1%)				0.8 (0.3-1.3)		0.8 (0-1.4)						0.8 (0.1-1.3)	
		Parent global (known for 117/203, 57.6%)				3.3 (0.7-5.4)		2.8 (1.0-6.0)						3.0 (0.9-5.7)	
		Physician global (known for 113/203, 55.7%)				3.0 (1.5-6.0)		2.0 (0.9-5.0)						2.5 (1.0-5.0)	
		ESR^h^ (known for 135/203, 66.5%)				17.0 (6.0-38.0)		22.5 (7.3-36.5)						20.2 (7.0-37.0)	
	**Medication, n (%)**														
		Methotrexate			31 (31)					39 (37.9)		70 (34.5)			
		Biologic			0 (0)					2 (1.9)		2 (1)			

^a^GCSE: General Certificate of Secondary Education, national exam taken at approximately age 16 years.

^b^Advanced Level: national exam taken at approximately age 18 years.

^c^HNC: higher national certificate.

^d^HND: higher national diploma.

^e^JIA: juvenile idiopathic arthritis.

^f^ERA: enthesitis-related arthritis.

^g^CHAQ: Child Health Assessment Questionnaire.

^h^ESR: erythrocyte sedimentation rate.

### Baseline Questionnaire Data

The PIP asks about child illness–related events; therefore, there were no normative data from parents of healthy children. Baseline PIP-F (mean 108.45, SE 2.14; mothers: mean 111.04, SE 2.31; fathers: mean 95.68, SE 5.22) and PIP-D scores (mean 102.84, SE 2.10; mothers: mean 104.73, SE 2.28; fathers: mean 93.48, SE 5.06) were worse than those reported by a sample of UK and US parents of children with a history of heart disease [31] (PIP-F mean 80.3 for mothers; mean difference 30.736, SE 2.306; *t*_7353301_=13.13; *P*<.001; and mean 76.2 for fathers; mean difference 19.483, SE 5.219; *t*_5175615_=3.73; *P*<.001); PIP-D mean 80.6 for mothers; mean difference 24.132, SE 2.284; *t*_1063948_=10.57; *P*<.001); and mean 75.7 for fathers; mean difference 17.776, SE 5.058; *t*_1806544_=3.51; *P*<.001) and UK parents of adolescents with chronic pain [32] (PIP-F mean 104.9; mean difference 3.554, SE 2.140; *t*_6166319_=1.66; *P*=.01) and PIP-D mean 98.0; mean difference 4.839, SE 2.097; *t*_569347_=2.31; *P*=.02).

Baseline scores for Hospital Anxiety and Depression Scale anxiety and depression (mean 9.04, SD 0.34, and mean 5.49, SD 0.29, respectively) were significantly worse than published UK normative data [33] (anxiety: mean 6.14, SD 3.76; depression: mean 3.68, SD 3.07; anxiety: mean difference 2.896, SE 0.341; *t*_62011_=8.49; *P*<.001; depression: mean difference 1.806, SE=0.285; *t*_196469_=6.33; *P*<.001). Of the 220 participants, scoring in the *mild* (score 8-10), *moderate* (11-14), or *severe* (15-21) ranges for anxiety were 48 (21.8%), 47 (21.4%), and 34 (15.5%) participants, respectively, and for depression were 43 (19.5%), 24 (10.9%), and 3 (1.4%) participants, respectively. This compares with 19% of the women and 12.5% of the men scoring in the moderate to severe ranges for anxiety and 6.9% of both men and women scoring in the moderate to severe ranges for depression in a UK normative sample [34].

Baseline PASE self-efficacy scores were mean 4.44 (SE 0.15) for symptoms (mothers: mean 4.42, SE 0.16; fathers: mean 4.54, SE 0.39) and mean 5.80 (SE 0.15) for psychosocial (mothers: mean 5.78, SE 0.16; fathers: mean 5.92, SE 0.14). These are approximately at the scale midpoint and are worse than those reported in the original scale validation [22] by mothers (symptoms: mean difference –0.428, SE 0.157; *t*_8485_=–2.73; *P*=.006; psychosocial: mean difference –0.620, SE 0.164; *t*_3078_=–3.77; *P*<.001) but not by fathers (symptoms: mean difference 0.681, SE 0.385; *t*_12222_=1.77; *P*=.08; psychosocial: mean difference –0.308, SE 0.414; *t*_6936_=–0.74; *P*=.46).

The total score on the ECS17-A was mean 77.87 (SE 0.97). This score reflects that, on average, parents felt that they could *usually* manage their child’s health care. The score on the Client Satisfaction Questionnaire of mean 28.4 (SE 0.24) reflects very high satisfaction with health services.

The CHQ-PF50 health-related quality of life summary scores of participants’ children were mean 33.4 (SE 0.95) for physical quality of life and mean 44.4 (SE 0.72) for psychosocial quality of life, which are poorer than the scores reported for UK healthy controls [25] (mean 55.4, SD 4.2; mean difference –21.999, SE 0.947; *t*_34421_=–23.23; *P*<.001 for physical quality of life; mean 51.6, SD 7.1; mean difference –7.199, SE 0.721; *t*_27490_=–9.98; *P*<.001 for psychosocial quality of life).

### Trial Outcomes

#### Overview

**Figure 2:**
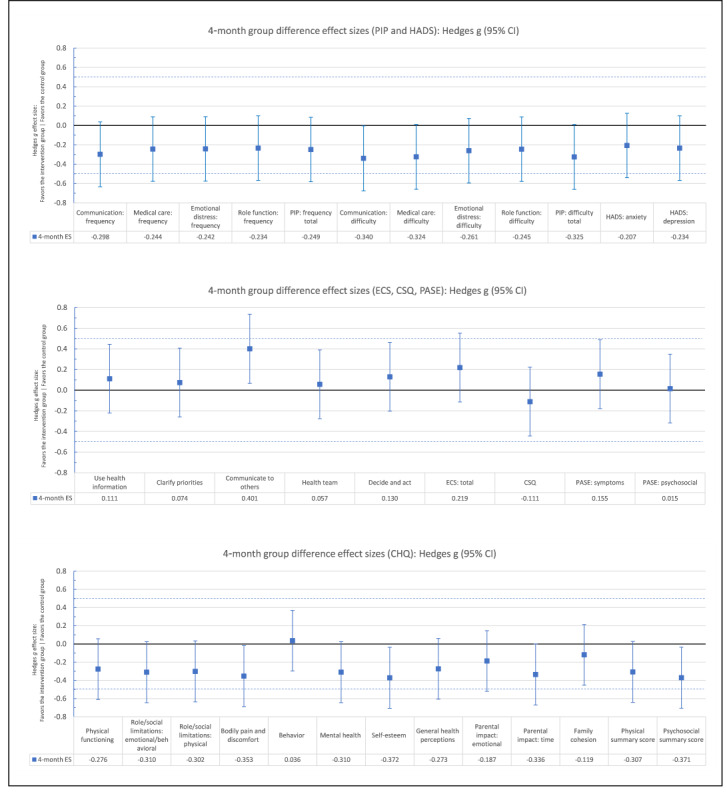
Group difference effect sizes at 4 months after randomization for all trial outcomes.CHQ: Child Health Questionnaire; CSQ: Client Satisfaction Questionnaire; ECS: Effective Consumer Scale; ES: effect size; HADS: Hospital Anxiety and Depression Scale; PASE: Parent’s Arthritis Self-Efficacy Scale; PIP: Pediatric Inventory for Parents.

**Figure 3:**
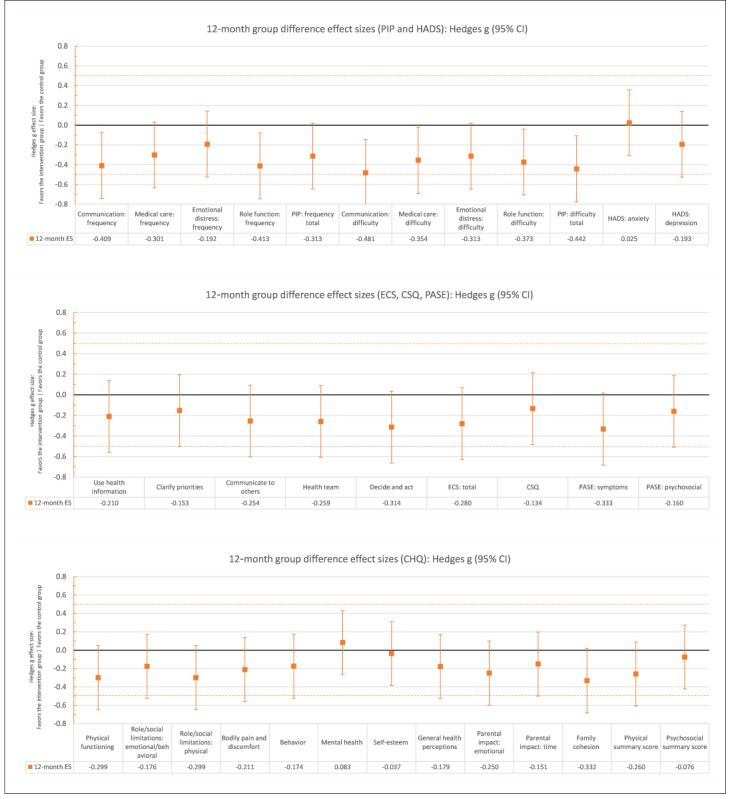
Group difference effect sizes at 12 months after randomization for all trial outcomes.CHQ: Child Health Questionnaire; CSQ: Client Satisfaction Questionnaire; ECS: Effective Consumer Scale; ES: effect size; HADS: Hospital Anxiety and Depression Scale; PASE: Parent’s Arthritis Self-Efficacy Scale; PIP: Pediatric Inventory for Parents

Tables 3 and 4 present adjusted means at each follow-up per group and multilevel modeling analyses estimates for the effect of trial arm and time and their interaction on all outcomes, adjusted for baseline scores and education level, respectively. The use of random effects for parent clusters was not possible because the number of dyad clusters was too few and random effects analyses did not converge. Consequently, parents were treated as individual units. Post hoc comparisons of trial arm effects at 4 months and 12 months are reported in Multimedia Appendix 2. The direction of effects, shown in Figures 2 and 3, mostly favored the intervention arm. Results for individual outcomes are reported in the next sections.

**Table 3:** Follow-up adjusted means (adjusted for baseline scores and educational level) on each outcome for the control and intervention groups in multilevel modeling analysis.

Variable			Control				Intervention		
			4 months, adjusted mean^a^ (95% CI)		12 months, adjusted mean (95% CI)		4 months, adjusted mean (95% CI)		12 months, adjusted mean (95% CI)
**PIP^b^frequency**									
	Communication	19.01 (17.86-20.16)		19.47 (18.27-20.68)		17.45 (16.16-18.74)		17.36 (16.05-18.67)	
	Medical care	18.29 (16.95-19.63)		18.48 (17.04-19.91)		16.80 (15.30-18.31)		16.63 (15.07-18.20)	
	Emotional distress	37.86 (35.65-40.07)		36.95 (34.68-39.21)		35.43 (32.95-37.90)		35.08 (32.60-37.57)	
	Role function	19.50 (18.24-20.76)		20.10 (18.77-21.42)		18.16 (16.75-19.57)		17.74 (16.28-19.21)	
	Frequency total	94.38 (89.04-99.73)		94.62 (89.13-100.11)		88.32 (82.32-94.31)		87.26 (81.27-93.24)	
**PIP** **difficulty**									
	Communication	17.64 (16.45-18.82)		18.13 (16.87-19.39)		15.82 (14.52-17.12)		15.57 (14.24-16.90)	
	Medical care	16.88 (15.53-18.23)		16.57 (15.21-17.93)		14.89 (13.37-16.41)		14.49 (12.98-16.00)	
	Emotional distress	38.87 (36.33-41.41)		38.47 (35.93-41.00)		35.85 (32.98-38.72)		35.08 (32.32-37.84)	
	Role function	19.15 (17.73-20.57)		19.39 (17.97-20.82)		17.57 (15.99-19.16)		17.10 (15.51-18.68)	
	Difficulty total	92.63 (86.86-98.40)		92.35 (86.77-97.93)		84.09 (77.60-90.58)		81.82 (75.76-87.87)	
**HADS^c^**									
	Anxiety	8.33 (7.57-9.09)		7.86 (7.01-8.71)		7.61 (6.75-8.46)		7.95 (7.02-8.87)	
	Depression	5.52 (4.82-6.21)		5.64 (4.93-6.35)		4.78 (4.00-5.57)		5.05 (4.27-5.84)	
**PASE^d^**									
	Symptoms	5.31 (4.92-5.69)		5.09 (4.65-5.52)		5.03 (4.57-5.49)		5.71 (5.24-6.18)	
	Psychosocial	6.58 (6.12-7.03)		6.31 (5.83-6.79)		6.55 (6.04-7.06)		6.64 (6.11-7.16)	
**ECS17-A^e^**									
	Use health information	76.70 (73.47-79.93)		78.32 (75.31-81.32)		75.07 (73.22-76.92)		81.04 (79.36-82.73)	
	Clarify priorities	82.89 (80.07-85.72)		83.65 (80.64-86.67)		81.94 (78.79-85.08)		85.62 (82.37-88.87)	
	Communicate with others	85.21 (82.29-88.14)		84.20 (81.32-87.08)		79.87 (78.19-81.55)		87.34 (85.74-88.94)	
	Negotiate roles	75.45 (72.12-78.79)		76.03 (72.63-79.42)		74.59 (70.85-78.32)		79.79 (76.10-83.49)	
	Decide and act	77.01 (73.82-80.20)		76.90 (73.97-79.83)		75.12 (71.53-78.70)		80.84 (77.64-84.04)	
	ESC17-A total	79.15 (76.66-81.64)		79.45 (76.90-82.01)		76.67 (73.88-79.46)		82.51 (79.73-85.29)	
**CSQ^f^**			28.30 (27.51-29.09)		28.07 (27.30-28.84)		28.70 (27.81-29.59)		28.51 (27.67-29.35)
**CHQ-PF50^g^**									
	Physical functioning	74.49 (69.83-79.15)		76.80 (71.94-81.66)		80.35 (75.10-85.61)		83.02 (77.73-88.30)	
	Role/social limitations–emotional/behavioral	80.79 (75.72-85.85)		83.62 (78.83-88.40)		87.94 (82.25-93.63)		87.22 (82.02-92.43)	
	Role/social limitations–physical	78.77 (73.63-83.92)		80.17 (75.03-85.31)		85.86 (80.06-91.66)		86.75 (81.16-92.35)	
	Bodily pain and discomfort	58.85 (54.06-63.63)		62.40 (57.02-67.77)		66.55 (61.16-71.94)		67.27 (61.40-73.14)	
	Behavior	67.15 (63.86-70.43)		67.19 (63.87-70.51)		66.61 (62.90-70.32)		69.66 (66.05-73.27)	
	Mental health	71.22 (68.40-74.04)		72.77 (69.46-76.08)		75.19 (72.03-78.34)		71.60 (68.01-75.20)	
	Self-esteem	70.06 (66.43-73.70)		76.42 (72.62-80.22)		76.23 (72.13-80.34)		77.02 (72.89-81.14)	
	General health perceptions	52.41 (49.31-55.52)		54.74 (51.39-58.09)		56.27 (52.78-59.77)		57.31 (53.65-60.97)	
	Parental impact–emotional	63.69 (58.95-68.44)		63.60 (58.99-68.20)		67.74 (62.38-73.10)		68.52 (63.50-73.54)	
	Parental impact–time	75.58 (70.84-80.32)		81.99 (77.65-86.34)		82.84 (77.49-88.19)		84.80 (80.10-89.51)	
	Family activities	71.69 (67.88-75.51)		73.47 (69.29-77.65)		77.01 (72.72-81.30)		79.09 (74.52-83.65)	
	Family cohesion	76.27 (72.09-80.44)		74.03 (69.89-78.17)		78.53 (73.81-83.24)		79.89 (75.41-84.37)	
	Physical summary scores	39.62 (37.14-42.09)		41.56 (38.90-44.22)		43.06 (40.32-45.80)		44.51 (41.63-47.40)	
	Psychosocial summary scores	45.78 (44.00-47.56)		48.06 (46.15-49.96)		48.79 (46.78-50.79)		48.68 (46.61-50.75)	

^a^Adjusted mean for baseline scores and educational level.

^b^PIP: Pediatric Inventory for Parents.

^c^HADS: Hospital Anxiety and Depression Scale.

^d^PASE: Parent’s Arthritis Self-Efficacy Scale.

^e^ECS17-A: Effective Consumer Scale–Adapted.

^f^CSQ: Client Satisfaction Questionnaire.

^g^CHQ-PF50: Child Health Questionnaire, 50-item parent version.

**Table 4:** Multilevel modeling analyses of each outcome exploring time and trial arm main effects and their interactions.

Variable					Effect					
					Trial arm		Time		Time×trial arm	
					*F* test (*df*)	*P* value	*F* test (*df*)	*P* value	*F* test (*df*)	*P* value
**PIP^a^ frequency**										
				Communication	5.37 (1,120627)	.02	0.01 (1,225948)	.90	0.32 (1,218596)	.58
				Medical care	2.87 (1,36383)	.09	0.04 (1,145323)	.84	0.01 (1,45835)	.75
				Emotional distress	1.17 (1,69328)	.28	0.07 (1,115020)	.79	0.10 (1,129609)	.75
				Role function	5.40 (1,27203)	.02	0.34 (1,89283)	.56	1.10 (1,67018)	.30
				Frequency total	3.14 (1,7872787)	.08	0.12 (1,2123768)	.73	0.09 (1,3941957)	.76
**PIP** **difficulty**										
				Communication	7.43 (1,2237)	.006	0.09 (1,6133)	.76	0.45 (1,5150)	.50
				Medical care	4.04 (1,2907)	.04	0.20 (1,1715)	.66	0.01 (1,6475)	.94
				Emotional distress	3.10 (1,6128)	.08	0.22 (1,7769)	.64	0.03 (1,15313)	.87
				Role function	4.37 (1,821)	.04	0.25 (1,1028)	.62	0.31 (1,1590)	.58
				Difficulty total	6.30 (1,588193)	.01	0.37 (1,115056)	.54	0.16 (1,194822)	.69
**HADS^b^**										
		Anxiety			0.02 (1,3123)	.89	0.56 (1,6920)	.45	1.72 (1,5892)	.19
		Depression			1.16 (1,11080)	.28	0.41 (1,4597)	.52	0.07 (1,12694)	.80
**PASE^c^**										
			Symptoms		3.63 (1,10281)	.06	4.80 (1,961)	.03	4.90 (1,7284)	.03
			Psychosocial		0.84 (1,7031)	.36	0.07 (1,12798)	.79	0.59 (1,3629)	.44
**ECS17-A^d^**										
	Use health information				1.42 (1,4.68E+09)	.23	9.90 (1,7.90E+10)	.002	2.89 (1,4.56E+08)	.09
	Clarify priorities				0.75 (1,1.95E+08)	.39	3.56 (1,8.41E+12)	.06	1.22 (1,3.55E+07)	.27
	Communicate with others				2.08 (1,2.03E+09)	.15	14.66 (1,2.07E+11)	<.001	10.33 (1,2.07E+08)	.001
	Negotiate roles				2.16 (1,1.40E+08)	.14	6.21 (1,1.33E+13)	.01	2.68 (1,5.77E+06)	.10
	Decide and act				3.17 (1,7.55E+06)	.08	8.63 (1,1.79E+08)	.003	4.91 (1,1.22E+07)	.03
	ECS17-A total				2.51 (1,2.29E+07)	.11	14.04 (1,7.75E+07)	<.001	6.90 (1,4.36E+06)	.009
**CSQ^e^**					0.57 (1,16736)	.45	0.16 (1,30483)	.69	0.00 (1,14841)	.95
**CHQ-PF50^f^**										
				Physical functioning	2.88 (1,546164)	.09	0.77 (1,403462)	.38	0.01 (1,533107)	.93
				Role/social limitations–emotional/behavioral	1.00 (1,360571)	.32	0.05 (1,454651)	.82	0.69 (1,577910)	.41
				Role/social limitations–physical	2.89 (1,273546)	.09	0.07 (1,359145)	.79	0.01 (1,66209)	.91
				Bodily pain and discomfort	1.44 (1,1039728)	.23	0.06 (1,579724)	.80	0.50 (1,777799)	.48
				Behavior	0.97 (1,37269)	.33	1.98 (1,48284)	.16	1.05 (1,28885)	.31
				Mental health	0.22 (1,96801)	.64	2.83 (1,264228)	.09	3.15 (1,83261)	.08
				Self-esteem	0.04 (1,22019)	.84	0.10 (1,92848)	.75	2.73 (1,71775)	.10
				General health perceptions	1.03 (1,198499)	.31	0.28 (1,353837)	.59	0.24 (1,174477)	.62
				Parental impact–emotional	2.00 (1,199245)	.16	0.07 (1,394803)	.79	0.05 (1,434994)	.82
				Parental impact–time	0.74 (1,650668)	.39	0.46 (1,434479)	.50	1.27 (1,237236)	.26
				Family activities	3.13 (1,186376)	.08	0.77 (1,396224)	.38	0.01 (1,493035)	.93
				Family cohesion	3.53 (1,225860)	.06	0.21 (1,222878)	.65	0.81 (1,321685)	.37
				Physical summary scores	2.13 (1,8378)	.14	0.93 (1,27479)	.33	0.06 (1,8741)	.81
				Psychosocial summary scores	0.19 (1,6310)	.66	0.01 (1,5005)	.93	2.31 (1,1647)	.13

^a^PIP: Pediatric Inventory for Parents.

^b^HADS: Hospital Anxiety and Depression Scale.

^c^PASE: Parent’s Arthritis Self-Efficacy Scale.

^d^ECS17-A: Effective Consumer Scale–Adapted.

^e^CSQ: Client Satisfaction Questionnaire.

^f^CHQ-PF50: Child Health Questionnaire, 50-item parent version.

#### Parenting Stress

A significant effect of the trial arm over the 2 follow-up periods was found on the PIP-F subscales *communication* and *role function* and on the PIP-D subscales *communication*, *medical care*, and *role function*, as well as the PIP-D total score. In each instance, participants in the intervention arm reported less frequency and difficulty of illness-related stressful events than participants in the control arm. Post hoc comparisons (Multimedia Appendix 2) found that these effects mostly reached statistical significance at 12 months. Effect sizes were small to medium.

#### Anxiety and Depression

We did not find a significant effect of the intervention on mean anxiety or depression scores.

#### Arthritis Self-efficacy

No significant effect of the trial arm was found on PASE. However, there was a significant time effect on PASE symptoms, where the whole sample improved over the 12-month period. In addition, there was a significant interaction effect on PASE symptoms: participants in the intervention arm reported greater improvement in their self-efficacy from 4 to 12 months than control participants.

#### Perceived Effectiveness in Managing Health Care

We did not find an overall effect of the trial arm on parents’ perceived effectiveness in managing their child’s health care assessed with the ECS17-A. We found significant interaction effects on the subscales *communicating with others* and *deciding and taking action*. For *communicating with others*, there was a significant trial arm effect at 4 months favoring the control arm, but the control arm did not change between 4 and 12 months, whereas the intervention arm improved significantly. For *deciding and taking action*, there was no effect of the trial arm at 4 or 12 months and the control arm did not change between 4 and 12 months, but the intervention arm improved significantly. We found a main effect of time on the subscales *use of health information* and *negotiating roles and taking control*, with an improvement in the overall sample on both subscales. There was no effect on the subscale *clarifying personal priorities*.

#### Satisfaction With Health Care

Satisfaction with health care services was very high at baseline and remained so at follow-ups with no significant differences between the trial arms.

#### Child’s Health-Related Quality of Life

There was no significant overall effect of the trial arm on parents’ assessment of their child’s health-related quality of life on the CHQ-PF50.

## Discussion

### Principal Findings

This RCT evaluated the *WebParC* website for parents of children with JIA. To our knowledge, this is the first website for parents of children with JIA that has undergone evaluation in an RCT. The website was found to be successful in reducing child illness–related parenting stress and also promoted a greater improvement in parents’ self-efficacy in managing children’s symptoms.

Although the direction of effects mostly favored the intervention, post hoc comparisons indicated that they did not reach statistical significance until 12 months. This suggests that it is in the longer term that the knowledge and skills parents gain from the website significantly reduce their stress and improve symptom self-efficacy.

Satisfaction with health care was very high among parents throughout this trial, indicating that even in the context of excellent clinical care, parents experience stress related to their child’s illness. This trial has shown that a web-based intervention, accessible when needed outside of the clinical setting, can help parents to manage the stress of having a child with JIA and could be offered to parents as an adjunct to the care given to their child. The effect sizes achieved ranged from small to medium, which is acceptable for a very *light touch* intervention that demands few additional resources.

Scores on the ECS17-A subscale *communicating with others* were high at all time points, reflecting a good degree of confidence in communicating with the health care team across the trial period. However, the intervention arm scores deteriorated at 4 months before improving again at 12 months. The drop at 4 months may indicate that access to the website meant that parents were less likely to engage with health care professionals in the early stage but had more interaction and had built up confidence in the longer term.

The parent outcomes improved by the website were those relating to the stress of communication; managing medical aspects of their child’s care, including symptoms; and carrying out everyday family and social roles. It is important that the website, which covers information about JIA and its treatment, including potentially distressing issues such as medication side effects, did not have any negative effect on parents’ psychological well-being. Of the 3 main *tasks* in living with a chronic illness proposed by Corbin and Strauss [35], two were improved by WebParC: medical management and role management. The third task, managing emotions, was unchanged.

We were unable to identify evaluations of other interventions specifically for parents of children with JIA. A review of interventions for parenting stress in families with pediatric conditions [35] did not include any web-based interventions. A Cochrane review of 47 psychological interventions for parents of children with chronic illnesses [37] included 6 interventions that were delivered at least partly on the web. Of these, only 2 small trials (n<40) assessed parental mental health; therapist-supported web-based family problem solving [38] for traumatic brain injury was found to be beneficial, but part–web-based cognitive behavioral therapy [39] did not have an effect on the mental health of parents who had a child with cancer. The primary target of WebParC was parenting stress rather than mental health; using web-based approaches to support the mental health of parents of children with JIA may require a greater focus on parents’ psychological well-being than we were able to achieve in WebParC.

Limitations of the study, in common with interventions of this type, include that it was not possible to blind participants to trial arm allocation. Although requested not to inform their child’s clinicians of their allocation, it is not possible to know whether all participants followed this request. We made every effort to ensure that where both parents participated, questionnaires were given to the individual parent for completion. Although we consider it unlikely, it is nonetheless possible that 1 parent completed both copies. However, the number of questionnaires received from both parents is small. A proportion of parents who consented to participate in the trial did not return the baseline questionnaire and were therefore not randomized. The follow-up response rates were also lower than expected. Parents may have forgotten or not prioritized questionnaire completion; when reminders and a small incentive were introduced midtrial, rates of baseline and follow-up questionnaire return improved. Another possibility is that although parents consented to the trial when they were at the clinic, taking part in research about their own well-being, rather than their child’s, was not a priority for them. Parents who did not return the baseline questionnaire may also have been reluctant to answer detailed questions about their own and their child’s well-being. These issues will need to be considered in future studies of this type.

In common with other research [40], fathers were less likely to participate, which occurred in this trial because they were less likely than mothers to attend the clinic. It was not possible to establish whether nonparticipating fathers may have been given access to the website by participating partners. The small number of cases where both parents participated meant that we were unable to cluster by household in our analyses.

To minimize participant burden, outcomes were assessed at only two follow-up times, 4 months and 12 months, after randomization. These were chosen for pragmatic reasons and to allow parents time to use the website before assessing its impact in the short and medium-to-longer term. We acknowledge that we will not have been able to capture all potential stressors that may have occurred and coping strategies used between the baseline and follow-up periods, but more frequent assessments would have increased the burden on parents.

Primary analyses with the PIP scales used a *P* value of <.05 for significance as per the protocol. For secondary analyses on additional scales we did not adjust the *P* value of <.05 to allow for multiple testing; therefore, caution should be taken when interpreting the results. However, it is notable that the pattern of our findings, even where not statistically significant, were mostly in the direction favoring the Intervention arm; therefore, it is unlikely that our significant findings reflect type I error.

### Conclusions

In conclusion, the study reported in this paper has demonstrated that web-based interventions for parents of children with JIA that combine information and skills training can result in significant benefits for parents. The benefits of reduced illness-related parenting stress and improvements in confidence and self-efficacy regarding parenting skills are important for a group not often seen as a high priority in health care. Future studies should attempt to devise techniques that reduce the loss to follow-up that was higher than projected in this study. In general, web-based interventions for parents of children with a chronic illness should be made a priority because they are easy to access at any time, replicable, and can offer a preventive approach to a large number of parents.

## Appendix

Multimedia Appendix 1 Unadjusted means and SEs for all self-report questionnaire measures at baseline, 4 months, and 12 months.

Multimedia Appendix 2 Post hoc comparisons of trial arm effects, adjusted for baseline scores and educational level.

Multimedia Appendix 3 CONSORT-EHeatlh V1.6.1.

## Abbreviations

CHQ-PF50: Child Health Questionnaire, 50-item parent version
ECS17-A: Effective Consumer Scale–Adapted
JIA: juvenile idiopathic arthritis
MCAR: missing completely at random
PASE: Parent’s Arthritis Self-Efficacy Scale
PIP: Pediatric Inventory for Parents
PIP-D: Pediatric Inventory for Parents, difficulty subscale
PIP-F: Pediatric Inventory for Parents, frequency subscale
RCT: randomized controlled trial
