# Supplementary material for: The Effects of a Web-Based Tool for Parents of Children With Juvenile Idiopathic Arthritis: Randomized Controlled Trial
Source: J Med Internet Res. 2022 May 12;24(5):e29787. doi: 10.2196/29787 (PMC9136652; doi:10.2196/29787)
Supplement: Multimedia Appendix 1 [file jmir_v24i5e29787_app1.docx]

Multimedia Appendix 1. Unadjusted means (SE) for all self-report questionnaire measures at baseline, 4 months and 12 months

| **Variable** | Control | | | | | | Intervention | | | | | |
| --- | --- | --- | --- | --- | --- | --- | --- | --- | --- | --- | --- | --- |
|  | Baseline (n=114) | | 4 months (n=79) | | 12 months (n=69) | | Baseline (n=106) | | 4 months (n=62) | | 12 months (n=59) | |
|  | Mean | Std. Error | Mean | Std. Error | Mean | Std. Error | Mean | Std. Error | Mean | Std. Error | Mean | Std. Error |
| **PIP Frequency** |  |  |  |  |  |  |  |  |  |  |  |  |
| Communication | 22.10 | 0.623 | 19.36 | 0.787 | 19.66 | 0.830 | 21.59 | 0.626 | 17.42 | 0.768 | 16.91 | 0.792 |
| Medical care | 22.29 | 0.731 | 18.97 | 0.910 | 18.70 | 0.967 | 21.20 | 0.698 | 16.57 | 0.847 | 15.72 | 0.854 |
| Emotional Distress | 44.15 | 1.212 | 39.26 | 1.664 | 37.93 | 1.635 | 41.42 | 1.228 | 34.62 | 1.525 | 33.64 | 1.494 |
| Role Function | 22.53 | 0.784 | 20.08 | 0.908 | 20.44 | 1.025 | 21.41 | 0.717 | 17.89 | 0.844 | 17.12 | 0.886 |
| PIP Frequency Total | 111.11 | 3.045 | 97.62 | 3.982 | 96.50 | 4.163 | 105.60 | 2.989 | 86.93 | 3.597 | 83.34 | 3.721 |
| **PIP Difficulty** |  |  |  |  |  |  |  |  |  |  |  |  |
| Communication | 18.88 | 0.647 | 17.69 | 0.769 | 17.99 | 0.857 | 18.82 | 0.615 | 16.03 | 0.713 | 15.68 | 0.673 |
| Medical care | 18.73 | 0.680 | 17.11 | 0.873 | 16.60 | 0.874 | 18.31 | 0.603 | 14.73 | 0.682 | 14.20 | 0.661 |
| Emotional Distress | 45.20 | 1.258 | 39.70 | 1.642 | 38.98 | 1.688 | 43.23 | 1.200 | 35.44 | 1.583 | 34.33 | 1.503 |
| Role Function | 21.57 | 0.779 | 19.51 | 0.957 | 19.59 | 1.004 | 20.93 | 0.743 | 17.35 | 0.825 | 16.76 | 0.804 |
| PIP Difficulty Total | 104.28 | 3.015 | 94.07 | 3.900 | 92.90 | 4.003 | 101.29 | 2.911 | 83.52 | 3.433 | 80.52 | 3.217 |
| **HADS Anxiety** | 9.37 | 0.481 | 8.71 | 0.565 | 8.30 | 0.626 | 8.68 | 0.483 | 7.39 | 0.633 | 7.41 | 0.618 |
| **HADS Depression** | 6.02 | 0.415 | 5.94 | 0.506 | 6.35 | 0.562 | 4.91 | 0.386 | 4.38 | 0.466 | 4.28 | 0.474 |
| **PASE Symptom** | 4.33 | 0.199 | 5.22 | 0.245 | 5.01 | 0.243 | 4.56 | 0.211 | 5.11 | 0.223 | 5.84 | 0.260 |
| **PASE Psychosocial** | 5.55 | 0.220 | 6.49 | 0.249 | 6.16 | 0.284 | 6.08 | 0.210 | 6.68 | 0.254 | 6.79 | 0.268 |
| **Effective Consumer Scale** |  |  |  |  |  |  |  |  |  |  |  |  |
| Use Health Info | 75.11 | 1.721 | 76.40 | 1.915 | 78.14 | 1.830 | 76.65 | 1.609 | 75.54 | 2.141 | 81.29 | 1.766 |
| Clarify Priorities | 82.61 | 1.572 | 82.45 | 1.604 | 83.09 | 1.694 | 84.71 | 1.225 | 82.39 | 1.704 | 86.02 | 1.579 |
| Communicate with others | 83.92 | 1.683 | 85.65 | 1.846 | 83.94 | 1.864 | 84.24 | 1.375 | 79.44 | 1.904 | 86.86 | 1.815 |
| Negotiate roles | 75.37 | 1.615 | 76.37 | 1.949 | 76.36 | 2.011 | 72.87 | 1.778 | 74.03 | 2.186 | 79.13 | 2.108 |
| Decide and act | 77.18 | 1.667 | 77.88 | 1.838 | 77.08 | 1.981 | 74.88 | 1.742 | 74.09 | 2.017 | 79.45 | 1.869 |
| ESC Total | 78.02 | 1.436 | 79.50 | 1.534 | 79.31 | 1.661 | 77.71 | 1.296 | 76.42 | 1.688 | 82.00 | 1.596 |
| **CSQ8** | 28.59 | 0.336 | 28.38 | 0.517 | 28.08 | 0.516 | 28.21 | 0.353 | 28.64 | 0.413 | 28.54 | 0.415 |
| **CHQ-PF50** |  |  |  |  |  |  |  |  |  |  |  |  |
| Physical Functioning | 60.05 | 2.947 | 72.67 | 3.224 | 76.88 | 3.316 | 60.76 | 3.051 | 81.78 | 2.402 | 83.90 | 2.687 |
| Role/Social Emotional/ Behavioral | 71.09 | 2.973 | 79.45 | 3.352 | 83.54 | 3.026 | 75.00 | 2.632 | 88.35 | 2.464 | 88.86 | 2.464 |
| Role/Social Physical | 63.22 | 2.897 | 77.07 | 3.352 | 79.17 | 3.516 | 65.40 | 3.231 | 87.78 | 2.296 | 88.14 | 2.242 |
| Bodily Pain and Discomfort | 45.99 | 2.583 | 57.98 | 3.257 | 61.69 | 3.548 | 46.99 | 2.540 | 68.07 | 2.629 | 68.31 | 2.685 |
| Behavior | 65.33 | 1.907 | 66.56 | 2.327 | 68.59 | 2.489 | 62.82 | 1.995 | 65.48 | 2.858 | 69.63 | 2.628 |
| Mental Health | 66.63 | 1.909 | 70.02 | 1.931 | 72.46 | 2.169 | 68.05 | 1.595 | 75.64 | 1.777 | 72.43 | 1.888 |
| Self Esteem | 68.28 | 1.913 | 68.31 | 2.325 | 75.42 | 2.423 | 70.17 | 1.990 | 77.40 | 2.219 | 78.78 | 2.242 |
| General Health Perceptions | 56.59 | 1.767 | 51.47 | 2.298 | 53.07 | 2.229 | 59.73 | 1.798 | 58.24 | 2.123 | 58.38 | 2.207 |
| Emotional Impact on Parent | 52.57 | 2.380 | 63.55 | 2.975 | 62.52 | 3.228 | 52.54 | 2.701 | 68.36 | 3.218 | 68.62 | 2.821 |
| Parental Impact - Time | 72.05 | 2.572 | 74.26 | 3.132 | 80.81 | 3.054 | 75.42 | 2.525 | 84.19 | 2.514 | 86.38 | 2.045 |
| Family Activities | 64.47 | 2.483 | 69.74 | 2.884 | 71.99 | 3.334 | 68.22 | 2.148 | 78.86 | 2.244 | 81.36 | 2.192 |
| Family Cohesion | 73.83 | 2.117 | 75.24 | 2.708 | 72.32 | 2.813 | 79.21 | 2.023 | 79.81 | 2.558 | 81.06 | 2.390 |
| CHQ - Physical Summary | 32.20 | 1.280 | 38.40 | 1.673 | 40.83 | 1.775 | 34.69 | 1.409 | 44.59 | 1.164 | 45.64 | 1.278 |
| CHQ - Psychosocial Summary | 44.17 | 1.020 | 45.26 | 1.249 | 47.94 | 1.317 | 44.65 | 1.024 | 48.82 | 1.189 | 49.24 | 1.157 |
