# Supplementary material for: The Effects of a Web-Based Tool for Parents of Children With Juvenile Idiopathic Arthritis: Randomized Controlled Trial
Source: J Med Internet Res. 2022 May 12;24(5):e29787. doi: 10.2196/29787 (PMC9136652; doi:10.2196/29787)
Supplement: Multimedia Appendix 2 [file jmir_v24i5e29787_app2.docx]

Multimedia Appendix 2. Post-hoc comparisons of trial arm effects, adjusted for baseline scores and educational level

|  | 4-months (n=141 returned questionnaires at follow-up) | | | | | | | | |
| --- | --- | --- | --- | --- | --- | --- | --- | --- | --- |
|  | Mean Difference | | | Statistics | | Effect size (ES) | | | |
| **Variable** | Estimate | Std. Error | T-Ratio | DF | *P* | Hedge's *g* | SE of ES | 95% LCI | 95% UCI |
| **PIP Frequency** |  |  |  |  |  |  |  |  |  |
| Communication Total | 1.558 | 0.885 | 1.76 | 771631 | .078 | -0.298 | 0.171 | -0.632 | 0.036 |
| Medical care Total | 1.486 | 1.034 | 1.44 | 5793729 | .151 | -0.244 | 0.170 | -0.577 | 0.090 |
| Emotional Distress Total | 2.436 | 1.705 | 1.43 | 1395352 | .153 | -0.243 | 0.170 | -0.576 | 0.091 |
| Role Function Total | 1.343 | 0.970 | 1.39 | 202442 | .166 | -0.235 | 0.170 | -0.568 | 0.099 |
| Frequency Total | 6.066 | 4.120 | 1.47 | 15645614 | .141 | -0.250 | 0.170 | -0.583 | 0.084 |
| **PIP Difficulty** |  |  |  |  |  |  |  |  |  |
| Communication Total | 1.815 | 0.897 | 2.02 | 19209 | *.043* | -0.341 | 0.171 | -0.676 | -0.006 |
| Medical care Total | 1.989 | 1.035 | 1.92 | 2318298 | .055 | -0.324 | 0.171 | -0.659 | 0.011 |
| Emotional Distress Total | 3.021 | 1.960 | 1.54 | 68998 | .123 | -0.261 | 0.170 | -0.595 | 0.073 |
| Role Function Total | 1.579 | 1.098 | 1.44 | 5297 | .150 | -0.245 | 0.170 | -0.579 | 0.088 |
| Difficulty Total | 8.539 | 4.440 | 1.92 | 928313 | .055 | -0.325 | 0.171 | -0.660 | 0.010 |
| **HADS** |  |  |  |  |  |  |  |  |  |
| Anxiety Total | 0.723 | 0.589 | 1.23 | 37057 | .220 | -0.208 | 0.170 | -0.542 | 0.125 |
| Depression Total | 0.732 | 0.538 | 1.36 | 105930 | .174 | -0.231 | 0.170 | -0.565 | 0.103 |
| **PASE** |  |  |  |  |  |  |  |  |  |
| Symptoms | 0.275 | 0.307 | 0.9 | 1025 | .370 | -0.152 | 0.170 | -0.485 | 0.181 |
| Psychosocial | 0.026 | 0.359 | 0.07 | 474 | .943 | -0.013 | 0.170 | -0.345 | 0.320 |
| **Effective Consumer Scale** |  |  |  |  |  |  |  |  |  |
| Use Health Info | 1.632 | 2.486 | 0.660 | 80492399 | .512 | -0.111 | 0.170 | -0.444 | 0.222 |
| Clarify Priorities | 0.958 | 2.175 | 0.440 | 4585610 | .660 | -0.074 | 0.170 | -0.407 | 0.259 |
| Communicate to others | 5.343 | 2.255 | 2.370 | 24332963 | *.018* | -0.401 | 0.171 | -0.736 | -0.065 |
| Health team | 0.865 | 2.567 | 0.340 | 1762272 | .736 | -0.057 | 0.170 | -0.389 | 0.276 |
| Decide and act | 1.892 | 2.458 | 0.770 | 2489469 | .441 | -0.130 | 0.170 | -0.463 | 0.203 |
| ESC Total | 2.486 | 1.913 | 1.300 | 740208 | .194 | -0.219 | 0.170 | -0.553 | 0.114 |
| **Client Satisfaction Questionnaire** | -0.403 | 0.610 | -0.660 | 78773 | .509 | 0.111 | 0.170 | -0.222 | 0.444 |
| **Child Health Questionnaire** |  |  |  |  |  |  |  |  |  |
| Physical Functioning | -5.864 | 3.600 | -1.630 | 158813 | .103 | 0.276 | 0.170 | -0.058 | 0.610 |
| Role/Social Emotional/Behavioral | -7.150 | 3.900 | -1.830 | 209655 | .067 | 0.310 | 0.171 | -0.024 | 0.645 |
| Role/Social Physical | -7.088 | 3.982 | -1.780 | 135768 | .075 | 0.302 | 0.171 | -0.032 | 0.637 |
| Bodily Pain and Discomfort | -7.701 | 3.691 | -2.090 | 689379 | *.037* | 0.353 | 0.171 | 0.018 | 0.688 |
| Behavior | 0.541 | 2.538 | 0.210 | 67472 | .831 | -0.036 | 0.170 | -0.369 | 0.297 |
| Mental Health | -3.966 | 2.167 | -1.830 | 187192 | .067 | 0.310 | 0.171 | -0.025 | 0.644 |
| Self Esteem | -6.170 | 2.817 | -2.190 | 56962 | *.029* | 0.372 | 0.171 | 0.037 | 0.707 |
| General Health Perceptions | -3.859 | 2.395 | -1.61 | 4079919 | .107 | 0.273 | 0.170 | -0.061 | 0.607 |
| Emotional Impact on Parent | -4.044 | 3.666 | -1.100 | 184404 | .270 | 0.187 | 0.170 | -0.146 | 0.520 |
| Parental Impact | -7.254 | 3.663 | -1.980 | 484658 | *.048* | 0.336 | 0.171 | 0.001 | 0.671 |
| Family Activities | -5.316 | 2.947 | -1.800 | 786250 | .071 | 0.306 | 0.171 | -0.028 | 0.641 |
| Family Cohesion | -2.260 | 3.225 | -0.700 | 1483219 | .483 | 0.119 | 0.170 | -0.214 | 0.452 |
| Physical Summary Scores | -3.443 | 1.901 | -1.810 | 11012 | .070 | 0.307 | 0.171 | -0.027 | 0.642 |
| Psychosocial Summary Scores | -3.003 | 1.380 | -2.180 | 7012 | *.030* | 0.371 | 0.171 | 0.036 | 0.706 |

| **Variable** | 12-months (n=128 returned questionnaires at follow-up) | | | | | | | | |
| --- | --- | --- | --- | --- | --- | --- | --- | --- | --- |
|  | Mean Difference | | | Statistics | | Effect size | | | |
|  | Estimate | Std. Error | T-Ratio | DF | p | Hedge's *g* | SE of ES | 95% LCI | 95% UCI |
| **PIP Frequency** |  |  |  |  |  |  |  |  |  |
| Communication Total | 2.108 | 0.910 | 2.32 | 121910 | *.021* | -0.409 | 0.179 | -0.760 | -0.058 |
| Medical care Total | 1.844 | 1.089 | 1.69 | 36273 | .090 | -0.300 | 0.178 | -0.649 | 0.049 |
| Emotional Distress Total | 1.865 | 1.726 | 1.08 | 69227 | .280 | -0.192 | 0.178 | -0.540 | 0.157 |
| Role Function Total | 2.352 | 1.012 | 2.32 | 27121 | *.020* | -0.412 | 0.179 | -0.763 | -0.060 |
| Frequency Total | 7.361 | 4.158 | 1.77 | 7842640 | .077 | -0.313 | 0.178 | -0.663 | 0.036 |
| **PIP Difficulty** |  |  |  |  |  |  |  |  |  |
| Communication Total | 2.561 | 0.940 | 2.72 | 2233 | ***.007*** | -0.482 | 0.180 | -0.834 | -0.129 |
| Medical care Total | 2.085 | 1.037 | 2.01 | 2908 | *.045* | -0.355 | 0.179 | -0.705 | -0.005 |
| Emotional Distress Total | 3.387 | 1.922 | 1.76 | 6129 | .078 | -0.312 | 0.178 | -0.662 | 0.037 |
| Role Function Total | 2.297 | 1.098 | 2.09 | 822 | *.037* | -0.374 | 0.179 | -0.725 | -0.024 |
| Difficulty Total | 10.536 | 4.197 | 2.51 | 588520 | *.012* | -0.442 | 0.179 | -0.794 | -0.090 |
| **HADS** |  |  |  |  |  |  |  |  |  |
| Anxiety Total | -0.086 | 0.645 | -0.13 | 3117 | .894 | 0.024 | 0.177 | -0.324 | 0.371 |
| Depression Total | 0.587 | 0.545 | 1.08 | 11072 | .281 | -0.192 | 0.178 | -0.540 | 0.156 |
| **PASE** |  |  |  |  |  |  |  |  |  |
| Symptoms | -0.622 | 0.327 | -1.9 | 10332 | .057 | 0.334 | 0.179 | -0.016 | 0.684 |
| Psychosocial | -0.331 | 0.360 | -0.92 | 7080 | .359 | 0.161 | 0.178 | -0.187 | 0.509 |
| **Effective Consumer Scale** |  |  |  |  |  |  |  |  |  |
| Use Health Info | -2.727 | 2.285 | -1.190 | Inf | .233 | 0.210 | 0.178 | -0.139 | 0.558 |
| Clarify Priorities | -1.967 | 2.270 | -0.870 | 193178753 | .386 | 0.153 | 0.178 | -0.195 | 0.501 |
| Communicate to others | -3.137 | 2.176 | -1.440 | 2072086064 | .149 | 0.254 | 0.178 | -0.095 | 0.603 |
| Health team | -3.766 | 2.564 | -1.470 | 141954908 | .142 | 0.259 | 0.178 | -0.090 | 0.608 |
| Decide and act | -3.945 | 2.217 | -1.780 | 7596270 | .075 | 0.314 | 0.178 | -0.036 | 0.664 |
| ESC Total | -3.056 | 1.928 | -1.580 | 23072523 | .113 | 0.280 | 0.178 | -0.069 | 0.629 |
| **Client Satisfaction Questionnaire** | -0.441 | 0.582 | -0.760 | 16743 | .449 | 0.134 | 0.178 | -0.214 | 0.482 |
| **Child Health Questionnaire** |  |  |  |  |  |  |  |  |  |
| Physical Functioning | -6.216 | 3.666 | -1.700 | 545301 | .090 | 0.299 | 0.178 | -0.050 | 0.649 |
| Role/Social Emotional/Behavioral | -3.608 | 3.614 | -1.000 | 360645 | .318 | 0.176 | 0.178 | -0.172 | 0.524 |
| Role/Social Physical | -6.584 | 3.875 | -1.700 | 274084 | .089 | 0.299 | 0.178 | -0.050 | 0.649 |
| Bodily Pain and Discomfort | -4.879 | 4.065 | -1.200 | 1042253 | .230 | 0.211 | 0.178 | -0.137 | 0.560 |
| Behavior | -2.470 | 2.512 | -0.980 | 37253 | .326 | 0.174 | 0.178 | -0.174 | 0.522 |
| Mental Health | 1.165 | 2.499 | 0.470 | 96704 | .641 | -0.083 | 0.177 | -0.430 | 0.265 |
| Self Esteem | -0.595 | 2.877 | -0.210 | 22014 | .836 | 0.037 | 0.177 | -0.311 | 0.385 |
| General Health Perceptions | -2.572 | 2.536 | -1.010 | 198892 | .310 | 0.179 | 0.178 | -0.169 | 0.527 |
| Emotional Impact on Parent | -4.929 | 3.482 | -1.420 | 199139 | .157 | 0.250 | 0.178 | -0.099 | 0.598 |
| Parental Impact | -2.813 | 3.282 | -0.860 | 651701 | .391 | 0.151 | 0.178 | -0.197 | 0.500 |
| Family Activities | -5.614 | 3.175 | -1.770 | 185757 | .077 | 0.314 | 0.178 | -0.036 | 0.663 |
| Family Cohesion | -5.859 | 3.120 | -1.880 | 225871 | .060 | 0.332 | 0.179 | -0.018 | 0.682 |
| Physical Summary Scores | -2.954 | 2.022 | -1.460 | 8379 | .144 | 0.260 | 0.178 | -0.089 | 0.609 |
| Psychosocial Summary Scores | -0.626 | 1.440 | -0.430 | 6307 | .664 | 0.076 | 0.177 | -0.271 | 0.424 |

*Abbreviations: DF – degrees of Freedom, SE of ES – standard error of Effect Size, 95% LCI – 95% Lower Confidence interval, 95% UCI – 95% Upper Confidence interval*
